# Retraction

**DOI:** 10.1080/21623945.2023.2187568

**Published:** 2023-03-24

We, the Editors and Publisher of the journal Adipocyte have retracted the following article:

Breast cancer-released exosomes trigger cancer-associated cachexia to promote tumor progression (DOI: 10.1080/21623945.2018.1551688)

Since publication, concerns have been raised about the integrity of the data in the article, alongside concerns about the integrity of the images and text overlap with previously published articles. When approached for an explanation, the authors checked their data and confirmed there are fundamental errors present and they agree to the retraction of this article. The authors apologise for this oversight.

We have been informed in our decision-making by our policy on publishing ethics and integrity and the COPE guidelines on retractions.

The retracted article will remain online to maintain the scholarly record, but it will be digitally watermarked on each page as ‘Retracted’.

